# Microbial biotechnology approaches to mitigating the deterioration of construction and heritage materials

**DOI:** 10.1111/1751-7915.12795

**Published:** 2017-08-03

**Authors:** Pilar Junier, Edith Joseph

**Affiliations:** ^1^ Laboratory of Microbiology Institute of Biology University of Neuchâtel 2000 Neuchâtel Switzerland; ^2^ Laboratory of Technologies for Heritage Materials Institute of Chemistry University of Neuchâtel 2000 Neuchâtel Switzerland; ^3^ Haute Ecole Arc Conservation Restauration 2000 Neuchâtel Switzerland

## Abstract

Microorganisms are the main engines of elemental cycling in this planet and therefore have a profound impact on both organic and mineral substrates. As such, past and present human‐made structures and cultural heritage can be negatively affected by microbial activity. Processes such as bioweathering (rocks and minerals), biodeterioration (organic substrates) or biocorrosion (metals) participate to the degradation or structural damage of construction and heritage materials. This structural damage can cause major economic losses (e.g. replacement of cast‐iron pipes in water distribution networks), and in the case of heritage materials, the entire loss of invaluable objects or monuments. Even though one can regard the influence of microbial activity on construction and heritage materials as negative, remarkably, the same metabolic pathways involved in degradation can be exploited to increase the stability of these materials.

Microorganisms are the main engines of elemental cycling in this planet and therefore have a profound impact on both organic and mineral substrates. As such, past and present human‐made structures and cultural heritage can be negatively affected by microbial activity. Processes such as bioweathering (rocks and minerals), biodeterioration (organic substrates) or biocorrosion (metals) participate to the degradation or structural damage of construction and heritage materials (Gadd, 2017). This structural damage can cause major economic losses (e.g. replacement of cast‐iron pipes in water distribution networks; Sarin *et al*., 2004); and in the case of heritage materials, the entire loss of invaluable objects or monuments (Ranalli *et al*., 2005; Gadd, 2017). Even though one can regard the influence of microbial activity on construction and heritage materials as negative, remarkably, the same metabolic pathways involved in degradation can be exploited to increase the stability of these materials (Table 1). By prolonging the life cycle of construction materials, microbial biotechnology can contribute directly to make our cities more sustainable. In addition, given the societal importance of cultural heritage, microbial biotechnology can help to preserve an important component of human legacy.

**Table 1:** Microbial metabolisms and effect (negative or positive) on construction and cultural heritage materials

Microbial metabolism	Negative effect	Positive effect
Sulfate reduction	Biocorrosion of iron and iron alloys (Dinh *et al*., 2004; Videla and Herrera, 2005)	Removal of black crust on stone artwork (Cappitelli *et al*., 2006; Polo *et al*., 2010)
Iron reduction	Biocorrosion of iron and iron alloys (Schutz *et al*., 2015)	Production of stable corrosion products via biogenic mineral precipitation (Cote *et al*., 2015; Comensoli *et al*., 2017)
Oxalogenesis	Mineral dissolution and rock weathering (Gadd *et al*., 2014)	Biological patination of metals (Joseph *et al*., 2012a,b, 2013)
Chemoorganotrophic respiration	Degradation of natural or synthetic carbon compounds	Carbonatogenesis in self‐healing concrete (Jonkers, 2011; Dhami *et al*., 2013)
		Removal of organic matter from frescoes (Ranalli *et al*., 2005; Bosch‐Roig *et al*., 2016)
Redox reactions with metals	Discoloration and deterioration of stained glass. Alteration of pigments (Bastian *et al*., 2010)	Biologically induced mineral formation (Cote *et al*., 2015; Comensoli *et al*., 2017)

Using microbial metabolisms for the safeguard of human‐made structures and cultural heritage offers both opportunities and challenges. A major advantage is compatibility with the treated substrate. For example, while the application of organic coatings to inorganic substrates is a common practice in the conservation–restoration of metal sculptures, these coatings create a physical barrier that has a different behaviour than the metal core and will eventually become inefficient. In the case of stonework, the use of consolidants and water repellents is controversial due to their non‐reversibility and limited long‐term performance, and some reports suggest that the treatment contributes to accelerated stone decay (De Muynck *et al*., 2010). In contrast, formation of biogenic minerals (biomineralization) that integrate into the natural corrosion patina formed on the metal substrate generates a compatible passivating layer with extended efficiency (Volkland *et al*., 2001; Joseph *et al*., 2012a). When applied to stonework, the process is dubbed biodeposition and involves microbiologically induced calcite precipitation (MICP; Adolphe *et al*., 1990; Rodriguez‐Navarro *et al*., 2003; De Muynck *et al*., 2010). Another important asset of biotechnological approaches is the possibility to combine those with chemical remediation methods. This has been exemplified in the removal of surface deposits from stonework using sulfate‐reducing bacteria, and its combination with further treatment using biocides to eliminate microorganisms contributing to biodeterioration (in this case algae and fungi; Polo *et al*., 2010). However, other examples show the risk of altering the dynamics of resident microbial communities by the use of biocides, as it is the case of uncontrolled microbial growth in the invaluable Lascaux cave paintings (Bastian *et al*., 2010).

In addition to remediatory treatments, many biotechnological approaches are attractive because of their preventive nature. A good example of this is the manufacturing of self‐healing materials. Self‐healing materials have an enormous potential specially under conditions requiring long‐term reliability and with poor accessibility to the infrastructure (Hager *et al*., 2010). Different strategies have been investigated in substrates such as metals, ceramics and polymers, and although the precise nature of the treatment will vary, the principle remains similar. Self‐healing is in all cases based on the generation of a mobile phase that closes the cracks in the substrate (Hager *et al*., 2010). In terms of biotechnology, the most advanced of those technologies involves concrete structures. Several types of applications have been proposed including biological mortar, crack remediation, bacterial concrete and self‐healing concrete (De Muynck *et al*., 2010; Jonkers *et al*., 2010; Jonkers, 2011; Seifan *et al*., 2016).

Using living microorganisms also creates challenges. Probably one of the most tangible and hard to solve is the negative perception of the general public towards microbes. In all the examples given in Table 1, it is noticeable that while a particular microbial metabolism can be exploited in a positive way, it is also deleterious for a different substrate. Science fairs oriented to the public, live demonstrations and involving the final user in the early phases of product development are probably the most effective ways to counteract this. Regardless of the microbial metabolic process under scrutiny, the most commonly cited challenge resides in the cost of biological treatments. For example, in the case of biodeposition it has been estimated that due to the price of constituents, this biological solution will never be competitive on a purely economical basis. Only in the case of self‐healing building materials, a significant added value can be expected from decreasing the needs for manual inspection and repair (De Muynck *et al*., 2010). Time is also a major concern tightly linked to the cost of the biological solutions. In this case, maintaining conditions permissive to microbial activity for several days to weeks could bear a large fraction of the total cost in a biological intervention. Providing suitable conditions or dealing with intrinsic limitations of the material (e.g. extreme alkaline pH such as in the case of concrete; De Muynck *et al*., 2010) occupies a large fraction of the efforts to translate technologies intro praxis. Safety is another concern as undesirable microbial growth within human‐made structures could offset the benefits of the solution. Also, regulatory barriers can impair the spread of a given technology and the transfer of technologies between different countries. Finally, issues in terms of upscaling of production and delivery of the microorganisms onto the surface for treatment are also barriers for the large‐scale transfer of technologies developed in the laboratory into the real world. There are encouraging examples of innovative solutions for some of these problems. For example, in the case of technologies using MICP three alternative venues have been explored, which include the identification of active extracellular metabolites to be applied directly on the substrate, the use of dead cells or cellular fractions, or the enhancement of the activity of resident microorganisms (Tiano *et al*., 1999; De Muynck *et al*., 2010). These alternatives are feasible given that MICP appears to be a general consequence of various microbial metabolisms, suggesting a significant potential for the stimulation of endogenous resident microbes (Jimenez‐Lopez *et al*., 2007). Likewise, the use of enzymes rather than living organisms has been suggested in biocleaning methods (Ranalli *et al*., 2005; Bosch‐Roig and Ranalli, 2014; Bosch‐Roig *et al*., 2016). In terms of delivery, the use of endospore‐forming Firmicutes was common in the case of biocementation technologies, but has been criticized in terms of the safety and the possible undesired reactivation of dormant cells on the substrate (Rodriguez‐Navarro *et al*., 2003). In the same way, the combination of microorganisms with specific delivery matrixes that provide conditions for the desired metabolism has been evaluated in the case of treatment of stonework and sulfate reduction (Cappitelli *et al*., 2007) or for the delivery in self‐healing materials (Ersan *et al*., 2015).

In summary, a better understanding of the complex link between microbial metabolism and biogeochemical cycling has had surprising consequences in our current take of microbial activity and its relationship to construction and heritage materials. A very active field of research has spurred from the possibilities offered by these technologies. One can expect that by dealing with the challenges posed, these technologies will help to capitalize in the untapped potential of nature most accomplished chemists (microorganisms) for the synthesis of inorganic components in an eco‐friendly manner. The latter is probably the most significant promise of this biotechnological approach.

## Conflict of Interest

None declared.
